# Supplementary material for: Efficient direct solar-to-hydrogen conversion by in situ interface transformation of a tandem structure
Source: Nat Commun. 2015 Sep 15;6:8286. doi: 10.1038/ncomms9286 (PMC4579846; doi:10.1038/ncomms9286)
Supplement: Supplementary Information — Supplementary Figure 1-5 and Supplementary References [file ncomms9286-s1.pdf]

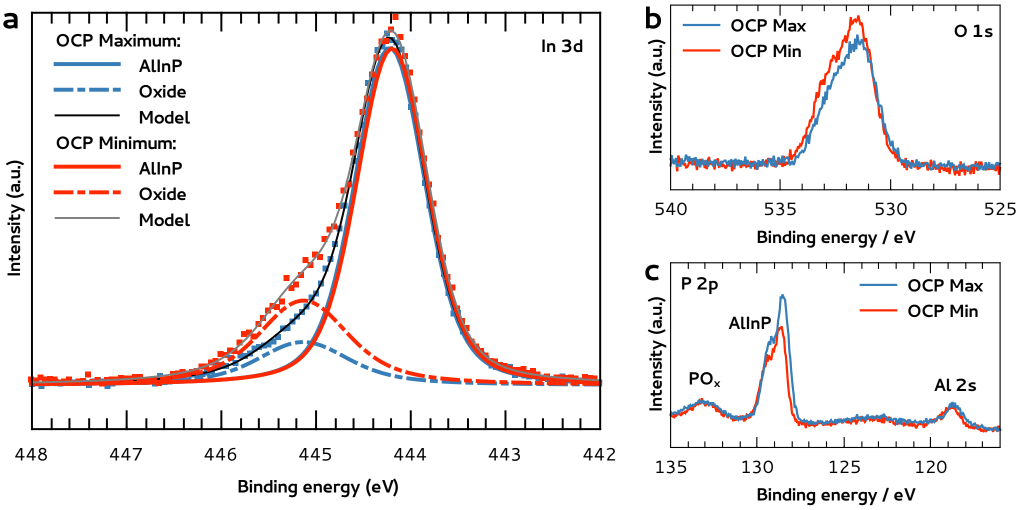

Supplementary Figure 1: **XPS at different stages of the OCP oscillation.** (a) The blue (red) curve shows the In 3d core level at an OCP maximum (minimum). The dashed lines indicate the oxide contribution. (b) O 1s core level. (c) P 2p and Al 2s core levels. To evaluate the oxide thickness, we used the model of a homogeneous overlayer of thickness d [1], assuming the same photoelectron cross sections for oxide and bulk. The electron attenuation length, λ, was calculated to be 2.5 nm [2], and the intensities of oxide and bulk are denoted I_o and I_b, respectively: $d = \lambda \ln\left(\frac{I_o}{I_b}+1\right)$. For the spectra displayed above, this results in an overlayer thickness of ca. (0.4 ± 0.1) nm for the OCP maximum and (0.8 ± 0.1) nm for the minimum.

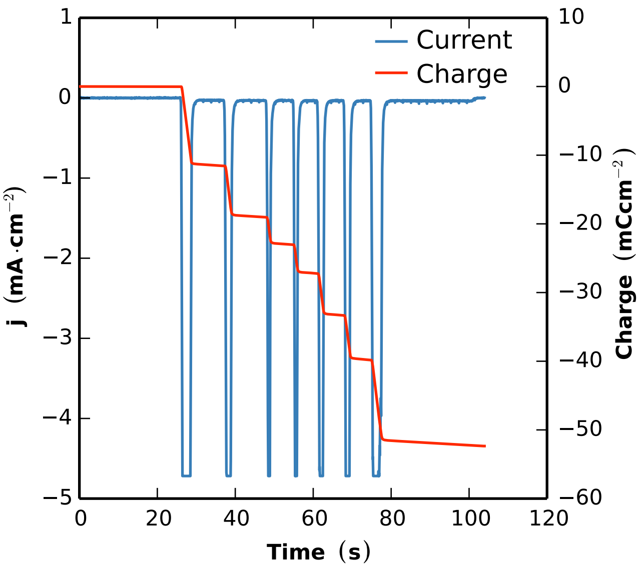

Supplementary Figure 2: **Photoelectrochemical catalyst deposition.** The blue curve shows the current density, the red curve the integrated charge.

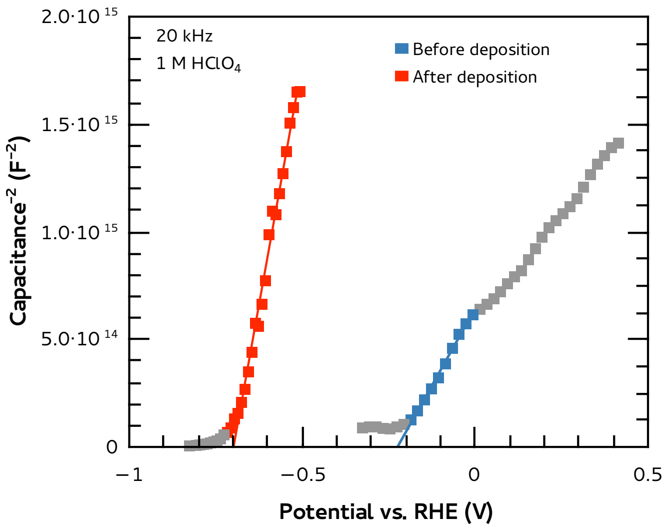

Supplementary Figure 3: **Evaluation of the flat-band potential.** The Mott-Schottky plots were measured in the dark after short OCP treatment (blue) and after Rh deposition (red). Linear fits show flat-band potentials of -0.22 V and -0.70 V vs. RHE before and after full surface transformation, respectively. Gray indicates data points that were not considered in the fit.

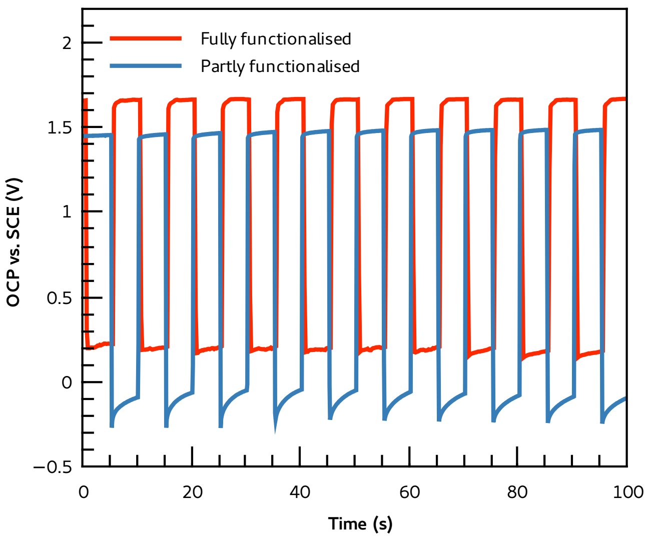

Supplementary Figure 4: **Open-circuit potential (OCP) transients.** The measurement employed a saturated calomel electrode (SCE) and chopped simulated AM 1.5G illumination for a partly (blue) and fully (red) functionalised sample. The overshoot of the partly functionalised sample indicates trapping of charge carriers in surface states [3]. The offset between both samples under blocked illumination arises due to very low residual illumination within the solar simulator setup, which was identical in both cases, demonstrating the higher photovoltage of the functionalised sample.

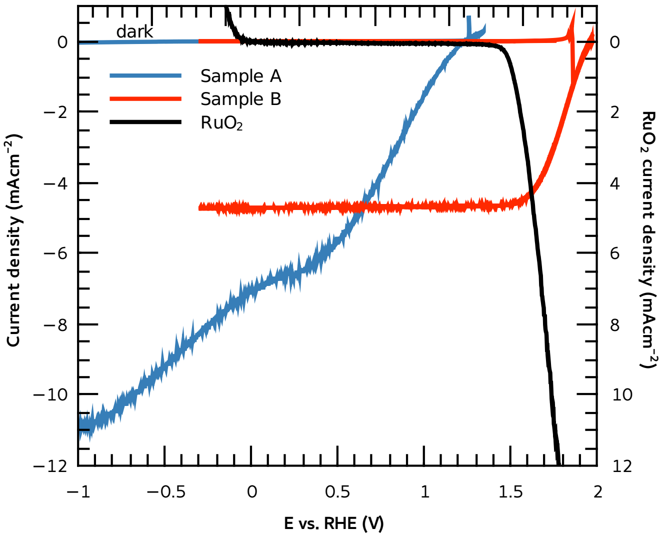

Supplementary Figure 5: **Comparison of differently functionalised samples.** Cyclic voltammograms under sim. sunlight for a sample without successful surface functionalisation (sample A, blue curve) reducing the fill factor and with catalyst overloading (sample B, red curve) reducing light transmission, but also recombination as evidenced by transient photocurrent analysis. The black curve shows the RuO_2 counter-electrode.

Supplementary References

[1] Briggs, D. & Seah, M. P. *Practical Surface Analysis*, vol. 1 (Wiley and Sons, Chichester, 1990).

[2] Powell, C. & Jablonski, A. NIST Electron Effective-Attenuation-Length Database, Version 1.3, Standard Reference Data Program Database 82, National Institute of Standards and Technology, Gaithersburg, MD (2011). URL `http://www.nist.gov/srd/nist82.cfm`.

[3] Li, J., Peat, R. & Peter, L. Surface recombination at semiconductor electrodes: Part II. Photoinduced "near-surface" recombination centres in p-GaP. *J. Electroanal. Chem.* **165**, 41–59 (1984).
